# Long-term Antibody Persistence After Hepatitis E Virus Infection and Vaccination in Dongtai, China

**DOI:** 10.1093/ofid/ofz144

**Published:** 2019-03-28

**Authors:** Brittany L Kmush, Huan Yu, Shoujie Huang, Xuefeng Zhang, Ting Wu, Kenrad E Nelson, Alain B Labrique

**Affiliations:** 1 Department of International Health, Johns Hopkins Bloomberg School of Public Health, Baltimore, Maryland; 2 National Institute of Diagnostics and Vaccine Development in Infectious Diseases, State Key Laboratory of Molecular Vaccinology and Molecular Diagnostics, School of Public Health, Xiamen University, Xiamen, China; 3 Jiangsu Provincial Center for Disease Control and Prevention, Nanjing, China; 4 Department of Epidemiology, Johns Hopkins Bloomberg School of Public Health, Baltimore, Maryland

**Keywords:** antibody persistence, epidemiology, hepatitis E vaccine, hepatitis E virus

## Abstract

**Background:**

Hepatitis E virus (HEV) is of global significance. HEV is a common cause of acute hepatitis in China. One of the major unanswered questions about HEV is the persistence of antibodies after infection and vaccination.

**Methods:**

We examined antibody persistence 6.5 years after HEV exposures through natural infection and vaccination. Ninety-seven vaccine recipients and 70 individuals asymptomatically infected with HEV enrolled in the phase III HEV239 vaccine trial in Dongtai, China, were revisited.

**Results:**

Antibody loss was 23.4% (95% confidence interval [CI], 17.1%–30.5%), with a nonsignificantly higher percentage of loss among those naturally infected (30.0%; 95% CI, 19.6%–42.1%) than those vaccinated (18.6%; 95% CI, 11.4%–27.7%; *P* = .085). Age and gender were not associated with antibody persistence. Only 2 people (1.2%) self-reported medically diagnosed jaundice or hepatitis-like illness in the last 10 years, both of whom had persistent antibodies. Contact with a jaundice patient and injectable contraceptive use were marginally associated with loss of detectable anti-HEV antibodies (*P* = .047 and .082, respectively), whereas transfusion was marginally associated with antibody persistence (*P* = .075).

**Conclusions:**

Antibody loss was more common among those naturally infected compared with those vaccinated. However, none of the characteristics examined were strongly associated with antibody loss, suggesting that factors not yet identified may play a more important role in antibody loss. Long-term postvaccination antibody persistence is currently unknown and will be an important consideration in the development of policies for the use of the highly efficacious HEV vaccine.

**ClinicalTrials.gov registration. ** NCT01014845.

Hepatitis E virus (HEV) causes approximately 20 million infections every year in developing countries. HEV usually causes acute hepatitis and is generally self-limiting, but can be very severe in pregnant women and immunocompromised patients [1–4]. In China, HEV is quite common, with seroprevalence estimates around 17% [5]. At least 9 epidemics of hepatitis E (HE) have been documented in China [6, 7], the largest of which occurred in 1986, with 122 000 cases reported and an overall case fatality rate of 0.87% [8]. Even though large epidemics of HEV have not been documented in China since the 1986 outbreak, HEV is an important cause of sporadic hepatitis and is estimated to cause about 20% of acute hepatitis cases [9].

In 2010, a large phase III trial of almost 100 000 participants was completed in Jiangsu Province, China, of a recombinant, subunit hepatitis E vaccine, HEV 239 [10]. This trial found the vaccine efficacy to be 100.0% (95% confidence interval [CI], 72.1%–100.0%) against clinical HE when all 3 doses were given. It was also found to be safe and well tolerated [10]. In December 2011, the HEV 239 vaccine, renamed Hecolin, was licensed by China’s State Food and Drug Administration, with production beginning in 2012 [11].

One of the major unanswered questions about HEV epidemiology is the persistence of antibodies, both after natural infection and after vaccination. Based on the paradigm established by other similar enteric pathogens, antibodies to HEV after an infectious episode were thought to be long lasting; however, a paucity of data exists to empirically support this assumption [12, 13]. The issue of antibody persistence has become of increasing importance due to mounting evidence of dramatically waning or absent antibody concentrations shortly after infection or vaccination. A number of studies have suggested that individuals can lose detectable antibodies to HEV, or “sero-revert.” In China, this phenomenon of sero-reversion has been observed in several cohorts. A large population-based study followed healthy, seropositive individuals for 1 year, during which time 1.4% of individuals experienced HEV sero-reversion [14]. Another large study found that 4.9% of seropositive participants had lost detectable antibodies after 2 years [15].

Antibody persistence after HEV vaccination has not been well characterized yet, due to the recent licensure of the vaccine. Follow-up of the vaccine trial participants suggests that vaccination provides protection against clinical HE, with 60 cases of HE identified 4.5 years after the trial concluded, 53 in the placebo group and only 7 in the experimental group [16]. During the phase II trial of HEV 239, the anti-HEV titers decreased by 76% only 6 months after the third dose of vaccine [17]. In a more recent follow-up of participants in the phase III HEV 239 vaccine trail, 76% of participants had detectable antibodies after 5 years [18]. Mathematical models of this data suggested that at least 50% of the participants would still have detectable antibodies anywhere from 8 to >30 years after vaccination, depending on the model used [18]. These studies suggest that in certain contexts, antibodies likely persist for a few years after exposure, but can also wane dramatically and, in some instances, become nondetectable. Whether this results in a renewed susceptibility to infection, or hepatitis E illness, is unclear and deserves further study, given the implications for the control of this important pathogen.

We examined risk factors associated with the persistence of antibodies after HEV infection and vaccination in a subset of individuals enrolled in the large phase III HEV vaccine trial in Dongtai, China. This is one of the first studies to explore the characteristics associated with long-term antibody persistence after HEV exposure in asymptomatic, naturally infected (with a known date of infection), and vaccinated individuals.

## METHODS

### Participant Selection and Enrollment

**Figure 1.**
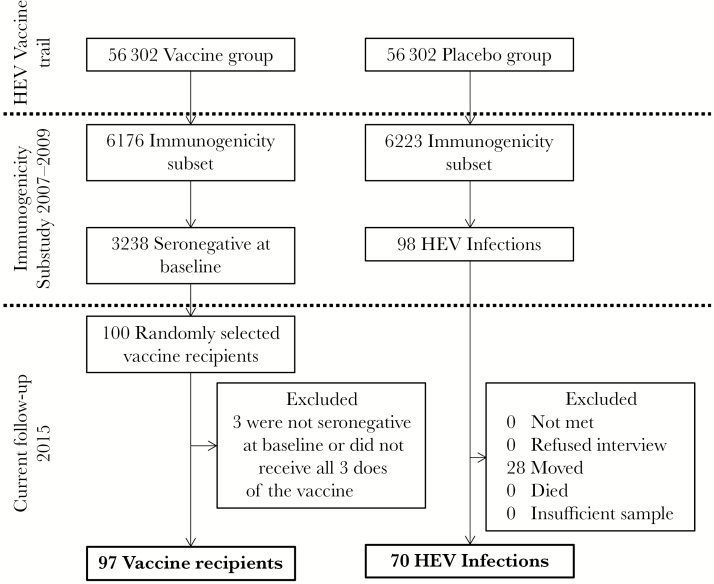
Cohort selection and follow-up diagram. Abbreviation: HEV, hepatitis E virus.

All participants in this study were recruited from a phase III clinical trial of a recombinant HEV vaccine. From 2007 to 2009, 112 604 men and women aged 16–65 years from Dongtai County, Jiangsu Province, China, were randomly assigned to receive either the experimental HEV vaccine or placebo (hepatitis B vaccine) [10]. We randomly selected 100 participants, stratified by age, to participate in this study from the immunogenicity subset of the vaccine trial. To be eligible for inclusion into this follow-up, the participants had to be anti-HEV IgG seronegative at baseline and had to have received all 3 doses of the vaccine (Figure 1). Of the 6223 participants who received all 3 doses of placebo in the immunogenicity subset, 98 individuals experienced a subclinical HEV infection, as assessed by HEV seroconversion between the baseline visit and 25 months after the third placebo dose (Figure 1) [19]. Blood was drawn at months 1, 13, and 25 after the full vaccination or placebo course to assess antibody status. These 98 individuals were asked to participate in this study.

In 2015, at 85 months after the full vaccination or placebo course, a 7-mL venous blood draw was taken from each participant, anthropometric measurements were taken (height, weight, and mid-upper arm circumference [MUAC]), and a detailed questionnaire assessing potential HEV exposures over the past 10 years was administered by trained study personnel. Shortly after the blood was drawn, the specimens were centrifuged, and the serum was separated and frozen at –80°C. All participants gave informed written consent before participation in the trial and all follow-up visits. The Ethics Committee of the Jiangsu Provincial Centre for Disease Control and Prevention approved all procedures for the vaccine trial, including ongoing follow-up. The trial is registered with ClinicalTrials.gov (NCT01014845). The Johns Hopkins School of Public Health Institutional Review Board also approved the study procedures described here.

### Hepatitis E Virus Antibody Testing

After the follow-up appointments were completed, serum samples were shipped to the National Institute of Diagnostics and Vaccine Development in Infectious Diseases in Xiamen, China. There, they were tested for anti-HEV IgG antibodies using an enzyme immune-assay (EIA) by Beijing Wantai Pharmacy Enterprise Co., Ltd. (Beijing, China). This EIA uses a segment of a recombinant ORF-2 protein and a solid phase indirect method for quantification of anti-HEV IgG antibodies [14]. This assay has been validated against a number of other ELISA assays, showing a greater degree of sensitivity than other commercially available assays [20, 21]. The manufacture’s cutoff of 0.077 Wu/mL was used to distinguish those who were anti-HEV positive from those who were anti-HEV negative.

### Statistical Methods

#### Risk Factor Assessment

Statistical analysis was performed using Stata, version 11 [22]. Characteristics between participants with a subclinical infection revisited for this antibody persistence follow-up and those lost to follow-up were compared using a Student *t* test for continuous variables or χ^2^ test for categorical variables. The vaccinated participants included in this follow-up were compared with the entire vaccinated immunogenicity cohort, published elsewhere [19]. After the anti-HEV testing was completed, each individual was assessed as positive or negative for anti-HEV IgG based on the manufacturer’s directions. Prevalence of antibody loss was calculated by dividing the number of participants negative for antibodies at follow-up by the total number of participants in this follow-up study. Antibody persistence was compared between natural infections and vaccine recipients using a χ^2^ test. Exposure and demographic risk factors were compared by antibody persistence status using a Student *t* test (continuous variables) or Fisher exact test (categorical variables) separately by vaccination status and overall. For the naturally infected participants, the midpoint between the seronegative time point and the seropositive time point was used as the time of seroconversion. For vaccine recipients, 1 month after receipt of the third vaccine dose was used as the time of seroconversion.

Nutritional status at the time of follow-up was determined using mid-upper arm circumference (MUAC), an indicator of chronic wasting [23]. Participants were also assessed as either having a normal MUAC or a low MUAC of <20 cm. From the height and weight measured by study personnel, body mass index (BMI) was calculated for each participant aged 20 years or older using the following formula: mass in kg/(height in m)^2^. BMI was also broken down into categories (<18.5 kg/m^2^: underweight; 18.5–25 kg/m^2^: normal; >25 kg/m^2^: overweight and obese).

Re-infection with hepatitis E was assessed by asking the participants if they were diagnosed with hepatitis or jaundice by a health care professional in the last 10 years. Although recall bias is an issue, we do not expect the recall to be different by antibody persistence status. Re-exposure to HEV was assessed in the subjects by asking about various water, sanitation, and animal exposures. We also asked if participants had contact with a jaundice patient in the last 10 years, as recent studies have suggested that this may be a potential route of exposure to HEV [24, 25]. As with recalling a hepatic illness, there is likely to be a great deal of recall bias, but it is not expected to differ by antibody persistence status. Possible blood-borne exposure routes assessed were self-reported injections and transfusions, as well as use of injected contraceptives in married females.

#### Regression Analysis

For the univariate analysis, Poisson regression with robust error variance was used to identify risk factors associated with antibody persistence [26]. Several multivariate models were developed using a Poisson regression with clustered robust error variance based on the results of the univariate analysis combined with previous scientific evidence from the literature. Overall models included all the participants adjusting for vaccination status, and stratified models separated those naturally infected from those who were vaccinated. Three models were developed: the first only included the demographic characteristics of age and gender. The second model added nutritional status. The third model included model 1 plus HEV exposure characteristics, including subsequent, self-reported hepatitis-like illness, contact with a jaundice patient, type of toilet, and household ownership of pigs, cows, goats or sheep, and chickens or ducks. Coefficients with a *P* value <.05 were considered significant. Model fit was assessed using the Bayesian Information Criterion (BIC).

## RESULTS

### Naturally Infected

We were able to revisit 70 of the 98 (71.4%) participants who experienced an asymptomatic, natural infection (Figure 1). The 28 participants who were not available for follow-up had all permanently moved out of the study area. The mean ages of the naturally infected participants revisited and those lost to follow-up were similar (53.2 vs 52.0 years, respectively; *P* = .570). Similar percentages of males and females were lost to follow-up (*P* = .300) (Table 1).

**Table 1.** Comparison Between Asymptomatic Hepatitis E Virus–Infected Participants Revisited and Lost to Follow-up, Dongtai, China (n = 98; 2015)

Characteristic	Revisited	Lost to Follow-up	*P* Value
No.	70	28	
Age at follow-up (SD), y	53.2 (9.41)	52.0 (10.3)	.570^a^
Gender, No. (%)			.300^b^
Male	27 (65.85)	14 (34.15)	
Female	43 (75.44)	14 (24.56)	

^a^Student *t* test.

^b^χ^2^ test.

In this study, we found 30% (21/70; 95% CI, 19.6%–42.1%) antibody loss among those naturally infected approximately 6 years after infection. Gender was not associated with antibody loss (*P* = .296). Injected contraceptive use in married women was higher among the sero-reverters (*P* = .031). Age at exposure did not differ between the 2 groups. MUAC was significantly smaller in those who were negative at follow-up than those who were positive at follow-up (28.4 vs 30.2 cm, respectively; *P* = .021). However, there was a very low prevalence of those with a low MUAC in this population, which did not differ by antibody status. BMI was also not different between the 2 groups. Only 2 participants reported medically diagnosed jaundice or hepatitis in the last 10 years, both of whom had persistent antibodies; this was not statistically significant. Contact with a jaundice patient was associated with sero-reversion (*P* = .026). However, both of these characteristics are based solely on participant recall, which is unreliable this long after exposure. Five participants had a blood transfusion in the last 10 years, all with persistent antibodies. A higher percentage of those negative at follow-up used tap water as their main source of drinking water, with the remainder using a tubewell (*P* = .040). However, the type of toilet used (sanitary vs unsanitary) did not differ by antibody persistence status (Supplementary Table 1).

### Vaccinated

We were able to revisit 100 out of the 100 randomly selected vaccinated participants. However, 3 of the participants in the vaccinated group were administered the questionnaire but were later found to not meet the inclusion criteria. They were either seropositive at baseline or did not receive all 3 doses of the vaccine. Only the 97 (97%) who met the inclusion criteria were included in this analysis. The mean ages of the participants were similar between the 2 groups (50.5 vs 51.3 years, respectively; *P* = .481). A somewhat higher percentage of males were included in this cohort (46.4%) than were included in the entire cohort (39.8%); however, this was not statistically significant (Table 2).

**Table 2.** Comparison Between Revisited Vaccinated Participants (n = 97) and Total Cohort of Vaccinated Participants, Dongtai, China (2015)

Characteristic	Revisited	Total Cohort [22]	*P* Value
No.	97	6176	
Age at follow-up (SD), y	50.5 (11.0)	51.3 (11.1)	.481^a^
Gender, No. (%)			.210^b^
Male	45 (46.39)	2457 (39.78)	
Female	52 (53.61)	3719 (60.22)	

^a^Student *t* test.

^b^χ^2^ test.

Among vaccinated participants, 18/97 (18.6%; 95% CI 11.4%–27.7%) no longer had detectable antibodies 6 years after vaccination. Vaccination status was marginally associated with antibody loss, 18.6% of the vaccinated participants were negative vs 30% negativity among the naturally infected participants (χ^2^*P* = .085). Age, gender, and nutritional status were not different by antibody persistence status. None of the vaccinated participants reported an incident of jaundice or hepatitis in the last 10 years. Five of the vaccinated participants had a transfusion in the last 10 years; all them were positive for antibodies at follow-up. Unsanitary toilet use was higher among those who no longer had detectable antibodies at follow-up (*P* = .019). However, no differences in drinking water source by antibody status were observed. Interestingly, family ownership of chickens or ducks was more common among those who were negative at follow-up (*P* = .043) (Supplementary Table 2).

### Overall

Antibody loss after about 6 years since exposure, either from infection or vaccination, was 23.4% (95% CI, 17.6%–30.5%) in this population (Table 3). Age and gender distribution did not differ by antibody persistence status. Markers of nutritional status, including BMI and MUAC, also did not differ between the groups. The majority of the participants fell into the normal BMI category (18.5–25 kg/m^2^; 59.9%) or overweight (25–30 kg/m^2^; 35.3%), with only a few participants considered underweight (<18.5 kg/m^2^; 1.8%) or obese (>30 kg/m^2^; 3.0%) (Table 3).

**Table 3.** Demographic and Exposure Risk Factors for Loss of Hepatitis E Virus Antibodies at Follow-up for the Entire Cohort in Dongtai, China (n = 167; 2015)

Characteristic	Positive at Follow-up		Negative at Follow-up		*P* Value
	(n = 128)		(n = 39)		
	Mean (SD)	Range	Mean (SD)	Range	Student *t* Test
Characteristic	Positive at Follow-up		Negative at Follow-up		*P* Value
	(n = 128)		(n = 39)		
Age at exposure, y	44.7 (12.5)	16.6–66.3	46.4 (12.1)	18.4–67.7	.4475
Time since exposure, y	6.56 (0.57)	5.44–7.00	6.44 (0.58)	5.44–7.00	.2566
BMI, kg/m^2^	24.5 (3.10)	14.2–33.3	24.7 (2.11)	21.5–29.8	.7763
MUAC, cm	29.6 (3.08)	20.4–38.0	29.1 (2.16)	22.5–32.0	.4125
	No.	%	No.	%	Fisher Exact Test
Vaccination status					.097
Asymptomatic infection	49	38.28	21	53.85	
Vaccinated	79	61.72	18	46.15	
Age at exposure, y					.855
16–19	5	3.91	1	2.56	
20–29	13	10.16	3	7.69	
30–39	23	17.97	8	20.51	
40–49	43	33.59	14	35.90	
50–59	29	22.66	6	15.38	
60–69	15	11.72	7	17.95	
Gender					.357
Male	58	45.31	14	35.9	
Female	70	54.69	25	64.1	
Pregnancy^a^					
Currently pregnant	1	1.47	0	0.00	1.00
No. times pregnant					.599
0	0	0.00	0	0.00	
1–3	67	95.71	22	91.67	
>3	3	4.29	2	8.33	
Nutritional status					
BMI, kg/m^2^					1.00
Underweight (<18.5)	3	2.34	0	0.00	
Normal (18.5–25)	76	59.38	24	61.54	
Overweight/obese (>25)	49	38.28	15	38.46	
MUAC, mm					1.00
Low MUAC (<22.5)	3	2.34	0	0.00	
Normal MUAC (≥22.5)	125	97.66	39	100.0	
Occupation					.238
Housework/none	13	10.16	4	10.26	
Farmer/fisherman/laborer	40	31.25	19	48.72	
Business owner	43	33.59	11	28.21	
Office-based service	29	22.66	4	10.26	
Other	3	2.34	1	2.56	
Type of work^b^					.145
Indoor	70	55.12	16	41.03	
Outdoor	57	44.88	23	58.97	
Subsequent jaundice/hepatitis					
Ever in the last 10 y	2	1.56	0	0.00	1.00
In the past 6 mo	0	0.00	0	0.00	N/A
In the past 6 mo to 1 y	1	0.78	0	0.00	.766
In the past 1 y to 10 y	1	0.78	0	0.00	.766
Contact with a person with jaundice (in the last 10 y)	3	2.34	4	10.26	.052
Injections (in the last 10 y)	128	100.0	39	100.0	N/A
Injected contraceptive use (in the last 1 y)^a^	20	28.57	12	50.00	.061
Blood transfusions (in the last 10 y)	10	7.81	0	0	.064
Drinking water source					.166
Tubewell	18	14.06	2	5.13	
Tap water	110	85.94	37	94.87	
Type of toilet					.067
	No.	%	No.	%	Fisher Exact Test
Unsanitary (open/hanging/pit)	53	41.41	23	58.97	
Sanitary (sealed/slab/flush)	75	58.59	16	41.03	
Hand washing					
Before eating	114	89.06	35	89.74	1.00
After defecation	120	93.75	36	92.31	.847
Eating outside the home					.365
Never	75	58.59	28	71.79	
<7 times/wk	38	29.69	8	20.51	
≥7 times/wk	15	11.72	3	7.69	
Animal owned by household					
Pig	7	5.47	3	7.69	.700
Cow	0	0.00	0	0.00	N/A
Goat/sheep	21	16.41	13	33.33	.039
Chicken/duck	19	14.84	11	28.21	.093
Rats^c^	66	51.56	26	66.67	.103

Abbreviations: BMI, body mass index; MUAC, mid-upper arm circumference.

^a^Calculated among married females only (n = 70 positive at follow-up; n = 24 negative at follow-up).

^b^One person positive at follow-up did not answer the question.

^c^Seen in household in the last 30 days.

Only 2 people (1.2%) self-reported a medically diagnosed jaundice or hepatitis in the last 10 years, both of whom had persistent antibodies. Injection use was very common in this group, with 100% of participants reporting receiving an injection within the last 10 years. A higher percentage of sero-reverting women reported using injected contraceptives; this was, however, not statistically significant (*P* = .061). Only 10 people reported having received a transfusion in the past decade, all of whom had persistent anti-HEV antibodies (*P* = .064) (Table 3).

In the univariate regression analysis (Table 4), contact with a jaundice patient, owning goats or sheep, and owning chickens or ducks statistically significantly increased the risk of antibody loss. However, the associations of animal ownership with antibody status were not seen in the multivariate analysis. Among the multivariate models (Table 4), model 1 with only demographic characteristics fit the best across the combined (Table 4) and stratified analyses (Supplementary Tables 3 and 4). Being underweight and a self-reported hepatitis-like illness (HLI) decreased the risk of antibody loss across the combined and stratified analyses. Among the vaccinated individuals, contact with a jaundice patient decreased the risk of antibody loss (Supplementary Table 4); the opposite association was seen in those with a natural infection (Supplementary Table 3). However, there were few people in each category, making many of these estimates unstable.

**Table 4.** Results of Univariate and Multivariate Poisson Regression Models for Risk Factors for Antibody Loss After Hepatitis E Virus Exposure in Dongtai, China (n = 167; 2015)

Characteristic	Univariate Analysis	Model 1^a^	Model 2^b^	Model 3^c^
Bayesian Information Criterion		–723.960	–715.392	–701.051
	RR (95% CI)	RR (95% CI)	RR (95% CI)	RR (95% CI)
Age at exposure (per 10 y)	1.09 (0.87–1.37)	1.08 (0.84–1.37)	1.10 (0.86–1.41)	1.01 (0.79–1.30)
Female gender	1.35 (0.76–2.42)	1.32 (0.74–2.35)	1.37 (0.77–2.44)	1.33 (0.75–2.37)
Vaccination	0.62 (0.37–1.07)	0.65 (0.37–1.12)	0.66 (0.38–1.14)	0.74 (0.42–1.31)
BMI,^d^ kg/m^2^				
Underweight (<18.5)	**1 × 10** ^**-6**^ **(3 × 10** ^**-7**^ **–4 × 10** ^**-6**^)		**2 × 10** ^**-6**^ **(5 × 10** ^**-7**^ **–6 × 10** ^**-6**^)	
Normal (18.5–25)	Ref.		Ref.	
Overweight/obese (>25)	0.98 (0.55–1.72)		1.05 (0.60–1.85)	
Subsequent HLI (last 10 y)	**1 × 10** ^**-6**^ **(3 × 10** ^**-7**^ **–5 × 10** ^**-6**^)			**3 × 10** ^**-7**^ **(5 × 10** ^**-8**^ **–1 × 10** ^**-6**^)
Contact with jaundice patient (last 10 y)	**2.61 (1.29–5.30)**			**2.35 (1.19–4.64)**
Sanitary toilet	0.58 (0.33–1.02)			0.66 (0.37–1.20)
Animal owned by household				
Pigs	1.31 (0.49–3.53)			0.87 (0.31–2.47)
Goat/sheep	**1.96 (1.13–3.39)**			1.36 (0.67–2.80)
Chicken or duck	**1.79 (1.00–3.19)**			1.43 (0.72–2.86)

Boldface indicates statistically significant results (*P* < .05).

Abbreviations: BMI, body mass index; CI, confidence interval; HLI, hepatitis-like illness; RR, relative risk.

^a^Model 1 (demographic characteristics) was adjusted for age, gender, and vaccination status.

^b^Model 2 (demographic + nutritional characteristics) was adjusted for model 1 plus body mass index.

^c^Model 3 (demographic + exposure characteristics) was adjusted for model 1 plus subsequent hepatitis-like illness, injections in the last 10 years, type of toilet, and household ownership of pigs, cows, goats or sheep, and chickens or ducks.

^d^In the univariate analysis, each category was tested against the reference category, adjusting for other categories.

## DISCUSSION

Overall antibody loss was 23.4% after 6.5 years, and none of the characteristics examined were strongly associated with antibody loss, suggesting that factors not yet identified may play a more important role in antibody loss after HEV infection and vaccination in this population. Antibody loss was greater, although not statistically significantly so, in those who experienced an asymptomatic natural infection compared with those who were vaccinated, with 30.0% vs 18.6% antibody loss in the 2 groups, respectively. This trend is consistent with previously published antibody kinetic studies from this population [18, 27]. However, this estimate of antibody loss after vaccination is lower than that previously reported after only 5 years of follow-up [18], suggesting that the 100 randomly selected vaccine recipients may not be representative of the entire cohort, despite similar age and gender distributions.

Age was not associated with persistent antibodies. A recent study from Bangladesh found that younger age at infection increased the risk of antibody loss [28]. However, children were excluded from the vaccine trial; thus, the full association of age at infection could not be examined here. Despite recent evidence that HEV may be transmitted from person to person [24, 25], sero-reverters were more likely to have had past contact with a jaundice patient than those who were positive at follow-up. Several studies have documented HEV transmission via transfusions or other methods of parenteral transmission [24, 29–31]. In this cohort, all 10 participants who reported receiving a blood transfusion in the last 10 years were positive at follow-up, suggesting that transfusion may pose a risk of exposure to HEV. Among those vaccinated, the use of a less sanitary toilet (open, hanging, pit vs sealed, slab, flush) and owning small livestock were more common among those who lost antibodies, a surprising association as these factors are considered sources of exposure to HEV. This suggests that characteristics other than re-exposure may be important for antibody persistence after vaccination. Among those naturally infected, self-reported contact with a jaundice patient was more common among those who had lost antibodies. However, this is an unreliable self-reported exposure that needs further investigation. Additionally, the use of the more sanitary municipal tap water was more common among those who had lost antibodies, suggesting that re-exposure to HEV may play a role in antibody persistence after natural infection.

One of the main limitations of this study is the small sample size, due to the limited number of individuals who experienced a documented asymptomatic infection. Furthermore, many of the estimates found in the multivariate analysis are unstable, due to the very small number of participants in each category. Additionally, it is impossible to tell if the antibodies observed in this follow-up remain from the original infectious episode or vaccination, or if they are from a subsequent infection. In recent years, asymptomatic and symptomatic reinfection with HEV has been documented [19]. Furthermore, it is unclear if the lack of detectable anti-HEV IgG antibodies is directly related to susceptibility to HEV infection. In hepatitis B virus infection, memory B cells, markers of immunological memory, are found in individuals with low or undetectable levels of circulating antibodies [32]. Recent evidence suggests that antibodies are correlated with B-cell markers of lasting immunity after HEV infection [33]. However, these correlates have not been well studied with HEV and could not be examined in this study. Future studies are needed to determine a definitive correlate of protection from future HEV infections. This is one of the first studies to compare long-term antibody persistence after HEV exposure between asymptomatic infected individuals with a known date of infection and vaccinated individuals and to examine characteristics associated with antibody persistence. Furthermore, both infected and vaccinated individuals were exposed to HEV within a short calendar time, eliminating the possibility of a cohort effect affecting antibody persistence. One of the difficulties in determining the persistence of antibodies after HEV infection has been the controversy over the accuracy of several available diagnostic tests [21, 34, 35]; however, only the well-validated, highly sensitive and specific Wantai assay was used to measure anti-HEV IgG in this study. Long-term postvaccination antibody persistence is currently unknown and will be a major factor in establishing policies that utilize the newly licensed vaccine.

## Supplementary Data

Supplementary materials are available at *Open Forum Infectious Diseases* online. Consisting of data provided by the authors to benefit the reader, the posted materials are not copyedited and are the sole responsibility of the authors, so questions or comments should be addressed to the corresponding author.

Supplementary_Materials
