# Erratum: Long-term Antibody Persistence After Hepatitis E Virus Infection and Vaccination in Dongtai, China

**DOI:** 10.1093/ofid/ofz224

**Published:** 2019-06-24

**Authors:** Brittany L Kmush, Huan Yu, Shoujie Huang, Xuefeng Zhang, Ting Wu, Kenrad E Nelson, Alain B Labrique

An error appeared in an article published in the April 2019 issue of the journal [Kmush BL, Yu H, Huang S, et al. Long-term Antibody Persistence After Hepatitis E Virus Infection and Vaccination in Dongtai, China. *Open Forum Infect Dis* 2019; 6(3):ofz144]. The author listing of “Xuefang Zhang” should state “Xuefeng Zhang.” This has now been corrected online.

