# Supplementary material for: Long-term Antibody Persistence After Hepatitis E Virus Infection and Vaccination in Dongtai, China
Source: Open Forum Infect Dis. 2019 Mar 28;6(4):ofz144. doi: 10.1093/ofid/ofz144 (PMC6475590; doi:10.1093/ofid/ofz144)
Supplement: Supplementary_Materials [file ofz144_suppl_supplementary_materials.docx]

# Supplemental Material

Supplemental Table 1: Demographic characteristics for asymptomatic hepatitis E virus infections in Dongtai, China (n = 70) (2015)

| **Characteristic** | **Positive at Follow-up**  **(n = 49)** | | **Negative at Follow-up**  **(n = 21)** | | **P-value** |
| --- | --- | --- | --- | --- | --- |
|  | **Mean (SD)** | **Range** | **Mean (SD)** | **Range** | **Student’s T-test** |
| Age at exposure (years) | 46.9 (9.06) | 18.5-62.9 | 48.1 (10.6) | 25.2-67.7 | 0.6197 |
| Time since exposure (years) | 5.96 (0.50) | 5.44-6.69 | 6.02 (0.47) | 5.44-6.69 | 0.6267 |
| BMI (kg/m^2^) | 24.9 (3.07) | 17.3-33.3 | 24.0 (1.65) | 21.5-27.4 | 0.2123 |
| MUAC (cm) | 30.2 (3.00) | 22.0-36.0 | 28.4 (2.28) | 22.5-32.0 | 0.0206 |
|  | **N** | **%** | **N** | **%** | **Fisher’s Exact Test** |
| Age at exposure (years) |  |  |  |  | 0.566 |
| 16-19 | 1 | 2.04 | 0 | 0.00 |  |
| 20-29 | 1 | 2.04 | 1 | 4.76 |  |
| 30-39 | 7 | 14.29 | 4 | 19.05 |  |
| 40-49 | 23 | 46.94 | 9 | 42.86 |  |
| 50-59 | 13 | 26.53 | 3 | 14.29 |  |
| 60-69 | 4 | 8.16 | 4 | 19.05 |  |
| Gender |  |  |  |  | 0.296 |
| Male | 21 | 44.86 | 6 | 28.57 |  |
| Female | 28 | 57.14 | 15 | 71.43 |  |
| Pregnancy* |  |  |  |  |  |
| Currently Pregnant | 0 | 0.00 | 0 | 0.00 | NA |
| No. times Pregnant |  |  |  |  | 1.00 |
| 0 | 0 | 0.00 | 0 | 0.00 |  |
| 1-3 | 27 | 96.43 | 13 | 92.86 |  |
| > 3 | 1 | 3.57 | 1 | 7.14 |  |
| Nutritional Status |  |  |  |  |  |
| BMI (kg/m^2^) |  |  |  |  | 0.599 |
| Underweight (< 18.5) | 1 | 2.04 | 0 | 0.00 |  |
| Normal (18.5-25) | 29 | 59.18 | 15 | 71.43 |  |
| Overweight/Obese (> 25) | 19 | 38.78 | 6 | 28.57 |  |
| MUAC (mm) |  |  |  |  | 1.00 |
| Low MUAC (< 22.5) | 1 | 2.04 | 0 | 0.00 |  |
| Normal MUAC (≥ 22.5) | 48 | 97.96 | 21 | 100.00 |  |
| Occupation |  |  |  |  | 0.265 |
| Housework/None | 6 | 12.24 | 1 | 4.76 |  |
| Farmer/Fisherman/Laborer | 18 | 36.73 | 12 | 57.14 |  |
| Business Owner | 12 | 24.49 | 6 | 28.57 |  |
| Office Based Service | 11 | 22.45 | 1 | 4.76 |  |
| Other | 2 | 4.08 | 1 | 4.76 |  |
| Type of work† |  |  |  |  | 0.436 |
| Indoor | 24 | 50.00 | 8 | 38.10 |  |
| Outdoor | 24 | 50.00 | 13 | 61.90 |  |
| Self-reported Jaundice/Hepatitis |  |  |  |  |  |
| Ever in the last 10 yrs. | 2 | 4.08 | 0 | 0.00 | 1.00 |
| in the past 6 mo. | 0 | 0.00 | 0 | 0.00 | NA |
| in the past 6 mo. to 1 yr. | 1 | 2.04 | 0 | 0.00 | 1.00 |
| in the past 1 yr. to 10 yrs. | 1 | 2.04 | 0 | 0.00 | 1.00 |
| Contact with a jaundice person  (in the last 10 years) | 1 | 2.04 | 4 | 19.05 | 0.026 |
| Injections (in the last 10 years) | 49 | 100.0 | 21 | 100.0 | NA |
| Injected Contraceptive use (in the last year)* | 8 | 28.57 | 9 | 60.00 | 0.031 |
| Blood transfusions (in the last 10 years) | 5 | 10.20 | 0 | 0.00 | 0.313 |
| Drinking Water Source |  |  |  |  | 0.040 |
| Tubewell | 17 | 34.69 | 2 | 9.52 |  |
| Tap water | 32 | 65.31 | 19 | 90.48 |  |
| Type of Toilet |  |  |  |  | 1.00 |
| Unsanitary (Open/hanging/pit) | 25 | 51.02 | 11 | 52.38 |  |
| Sanitary (sealed/slab/flush) | 24 | 48.98 | 10 | 47.62 |  |
| Hand washing |  |  |  |  |  |
| Before eating | 40 | 81.63 | 18 | 85.71 | 1.00 |
| After defecation | 43 | 87.76 | 19 | 90.48 | 1.00 |
| Eating outside the home |  |  |  |  | 0.657 |
| Never | 25 | 51.02 | 13 | 31.90 |  |
| < 7 times/week | 18 | 36.73 | 5 | 23.81 |  |
| ≥ 7 times/week | 6 | 12.24 | 3 | 14.29 |  |
| Animal owned by household |  |  |  |  |  |
| Pig | 6 | 12.24 | 2 | 9.52 | 1.00 |
| Cow | 0 | 0.00 | 0 | 0.00 | NA |
| Goat/Sheep | 12 | 24.49 | 8 | 38.10 | 0.263 |
| Chicken/Duck | 12 | 24.49 | 6 | 28.57 | 0.770 |
| Rats‡ | 29 | 59.18 | 17 | 80.95 | 0.103 |

*Calculated among married females only (n = 28 positive at follow-up; n = 14 negative at follow-up).

†1 person positive at follow-up did not answer the question.

‡Seen in household in the last 30 days.

Supplemental Table 2: Demographic characteristics for hepatitis E virus vaccine recipients in Dongtai, China (n = 97) (2015)

| **Characteristic** | **Positive at Follow=up**  **(n = 79)** | | **Negative at Follow-up**  **(n = 18)** | | **P-value** |
| --- | --- | --- | --- | --- | --- |
|  | **Mean (SD)** | **Range** | **Mean (SD)** | **Range** | **Student’s T-test** |
| Age at exposure (years) | 43.4 (14.1) | 16.6-66.1 | 44.5 (13.8) | 18.4-64.2 | 0.7653 |
| Time since exposure (years) | 6.94 (0.02) | 6.92-6.99 | 6.94 (0.02) | 6.93-6.99 | 0.8932 |
| BMI (kg/m^2^) | 24.3 (3.11) | 14.2-32.8 | 25.5 (2.35) | 27.8-29.8 | 0.1367 |
| MUAC (cm) | 29.2 (3.10) | 20.4-38 | 29.9 (1.73) | 27.0-32 | 0.3238 |
|  | **N** | **%** | **N** | **%** | **Fisher’s Exact Test** |
| Age at exposure (years) |  |  |  |  | 1.00 |
| 16-19 | 4 | 5.06 | 1 | 5.56 |  |
| 20-29 | 12 | 15.19 | 2 | 11.11 |  |
| 30-39 | 16 | 20.25 | 4 | 22.22 |  |
| 40-49 | 20 | 25.32 | 5 | 27.78 |  |
| 50-59 | 16 | 20.25 | 3 | 16.67 |  |
| 60-69 | 11 | 13.92 | 3 | 16.67 |  |
| Gender |  |  |  |  | 1.00 |
| Male | 37 | 46.84 | 8 | 44.44 |  |
| Female | 42 | 53.16 | 10 | 55.56 |  |
| Pregnancy* |  |  |  |  |  |
| Currently Pregnant | 1 | 2.50 | 0 | 0.00 | 1.00 |
| No. times Pregnant |  |  |  |  | 0.481 |
| 0 | 0 | 0.00 | 0 | 0.00 |  |
| 1-3 | 40 | 95.24 | 9 | 90.00 |  |
| > 3 | 2 | 4.76 | 1 | 10.00 |  |
| Nutritional Status |  |  |  |  |  |
| BMI (kg/m)^2^ |  |  |  |  | 0.625 |
| Underweight (< 18.5) | 2 | 2.53 | 0 | 0.00 |  |
| Normal (18.5-25) | 47 | 59.49 | 9 | 50.00 |  |
| Overweight/Obese (> 25) | 30 | 37.97 | 9 | 50.00 |  |
| MUAC (mm) |  |  |  |  | 1.00 |
| Low MUAC (< 22.5) | 2 | 2.53 | 0 | 0.00 |  |
| Normal MUAC (≥ 22.5) | 77 | 97.47 | 18 | 100.00 |  |
| Occupation |  |  |  |  | 0.593 |
| Housework/None | 7 | 8.86 | 3 | 16.67 |  |
| Farmer/Fisherman/Laborer | 22 | 27.85 | 7 | 38.89 |  |
| Business Owner | 31 | 39.24 | 5 | 27.78 |  |
| Office Based Service | 18 | 22.78 | 3 | 16.367 |  |
| Other | 1 | 1.27 | 0 | 0.00 |  |
| Type of work |  |  |  |  | 0.307 |
| Indoor | 46 | 58.23 | 8 | 44.44 |  |
| Outdoor | 33 | 41.77 | 10 | 55.56 |  |
| Self-reported Jaundice/Hepatitis |  |  |  |  |  |
| Ever in the last 10 yrs. | 0 | 0.00 | 0 | 0.00 | NA |
| in the past 6 mo. | 0 | 0.00 | 0 | 0.00 | NA |
| in the past 6 mo. to 1 yr. | 0 | 0.00 | 0 | 0.00 | NA |
| in the past 1 yr. to 10 yrs. | 0 | 0.00 | 0 | 0.00 | NA |
| Contact with a jaundice person  (in the last 10 years) | 2 | 2.53 | 0 | 0.00 | 1.00 |
| Injections (in the last 10 years) | 79 | 100.0 | 18 | 100.0 | NA |
| Injected Contraceptive use (in the last year)* | 12 | 30.77 | 3 | 30.00 | 1.00 |
| Blood transfusions (in the last 10 years) | 5 | 6.33 | 0 | 0.00 | 0.580 |
| Drinking Water Source |  |  |  |  | 1.00 |
| Tubewell | 1 | 1.27 | 0 | 0.00 |  |
| Tap water | 78 | 98.73 | 18 | 100.00 |  |
| Type of Toilet |  |  |  |  | 0.019 |
| Unsanitary (Open/hanging/pit) | 28 | 35.44 | 12 | 66.67 |  |
| Sanitary (sealed/slab/flush) | 51 | 64.56 | 6 | 33.33 |  |
| Hand washing |  |  |  |  |  |
| Before eating | 74 | 93.67 | 17 | 94.44 | 0.719 |
| After defecation | 77 | 97.47 | 17 | 94.44 | 0.464 |
| Eating outside the home |  |  |  |  | 0.222 |
| Never | 50 | 63.29 | 15 | 83.33 |  |
| < 7 times/week | 20 | 25.32 | 3 | 13.61 |  |
| ≥ 7 times/week | 9 | 11.39 | 0 | 0.00 |  |
| Animal owned by household |  |  |  |  |  |
| Pig | 1 | 1.27 | 1 | 5.56 | 0.338 |
| Cow | 0 | 0.00 | 0 | 0.00 | NA |
| Goat/Sheep | 9 | 11.39 | 5 | 27.78 | 0.129 |
| Chicken/Duck | 7 | 8.86 | 5 | 27.78 | 0.043 |
| Rats† | 37 | 46.84 | 9 | 50.00 | 1.00 |

*Calculated among married females only (n = 42 positive at follow-up; n = 10 negative at follow-up).

†Seen in household in the last 30 days.

Supplemental Table 3: Results of multivariate Poisson regression models for risk factors for antibody loss after asymptomatic hepatitis E virus infection in Dongtai, China (n = 70) (2015)

| **Characteristic** | **Model 1*** | **Model 2†** | **Model 3‡** |
| --- | --- | --- | --- |
| Bayesian Information Criterion | -235.323 | -228.105 | -216.144 |
|  | **RR (95% CI)** | **RR (95% CI)** | **RR (95% CI)** |
| Age at exposure (per 10 years) | 1.15 (0.76, 1.74) | 1.13 (0.75, 1.70) | 1.18 (0.72, 1.94) |
| Female Gender | 1.63 (0.72, 3.66) | 1.69 (.075, 3.79) | 1.95 (0.86, 4.39) |
| BMI (kg/m^2^) |  |  |  |
| Underweight (< 18.5) |  | **2x10^-6^ (2x10^-7^,1x10^-5^)** |  |
| Normal (18.5-25) |  | Ref. |  |
| Overweight/Obese (> 25) |  | 0.70 (0.31, 1.58) |  |
| Subsequent HLI (last 10 years) |  |  | **6x10^-7^ (9x10^-8^, 4x10^-6^)** |
| Contact with jaundice patient  (last 10 years) |  |  | **3.73 (1.80, 7.71)** |
| Sanitary Toilet |  |  | 1.12 (0.55, 2.29) |
| Animal owned by household |  |  |  |
| Pigs |  |  | 0.54 (0.15, 1.98) |
| Goat/Sheep |  |  | 1.62 (0.51, 5.14) |
| Chicken or Duck |  |  | 0.96 (0.31, 2.98) |

Boldface results represent statistically significant results (p < 0.05).

*Model 1 (demographic characteristics) is adjusted for age and gender.

†Model 2 (demographic + nutritional characteristics) is adjusted for model 1 plus body mass index (BMI).

‡Model 3 (demographic + exposure characteristics) is adjusted for model 1 plus subsequent hepatitis-like illness (HLI), injections in the last ten years, type of toilet and household owner ship of pigs, cows, goats or sheep, and chickens or ducks.

Supplemental Table 4: Results of multivariate Poisson regression models for risk factors for antibody loss after hepatitis E virus vaccination in Dongtai, China (n = 97) (2015)

| **Characteristic** | **Model 1*** | **Model 2†** | **Model 3‡** |
| --- | --- | --- | --- |
| Bayesian Information Criterion | -369.492 | -362.1421 | -354.085 |
|  | **RR (95% CI)** | **RR (95% CI)** | **RR (95% CI)** |
| Age at exposure (per 10 years) | 1.05 (0.78, 1.41) | 1.13 (0.83, 1.56) | 0.93 (0.67, 1.29) |
| Female Gender | 1.09 (0.45, 2.53) | 1.19 (0.51, 2.77) | 0.94 (0.38, 2.30) |
| BMI (kg/m^2^) |  |  |  |
| Underweight (< 18.5) |  | **1x10^-6^ (3x10^-7^, 7x10^-6^)** |  |
| Normal (18.5-25) |  | Ref. |  |
| Overweight/Obese (> 25) |  | 1.62 (0.68, 3.87) |  |
| Subsequent HLI (last 10 years) |  |  | Omitted |
| Contact with jaundice patient  (last 10 years) |  |  | **8x10^-7^ (2x10^-7^, 4x10^-6^)** |
| Sanitary Toilet |  |  | 0.38 (0.13, 1.11) |
| Animal owned by household |  |  |  |
| Pigs |  |  | 1.53 (0.46, 4.78) |
| Goat/Sheep |  |  | 1.09 (0.31, 3.83) |
| Chicken or Duck |  |  | 2.04 (0.77, 5.40) |

Boldface results represent statistically significant results (p < 0.05).

*Model 1 (demographic characteristics) is adjusted for age and gender.

†Model 2 (demographic + nutritional characteristics) is adjusted for model 1 plus body mass index (BMI).

‡Model 3 (demographic + exposure characteristics) is adjusted for model 1 plus subsequent hepatitis-like illness (HLI), injections in the last ten years, type of toilet and household owner ship of pigs, cows, goats or sheep, and chickens or ducks.
